# Geographic review on the specimens of the Caatinga Biome in the Jardim Botânico do Rio de Janeiro (RB) herbarium

**DOI:** 10.3897/BDJ.7.e38248

**Published:** 2019-10-03

**Authors:** Ulises Rodrigo Magdalena, Luís Alexandre Estevão da Silva, Felipe Alves Oliveira, Rafael Oliveira Lima, Ernani Bellon, Rafael Ribeiro, João Monnerat Lanna, Maria Luiza Abieri, Gabriel de Oliveira Cardoso, Aline Vieira Amorim de Amorim, Rafaela Campostrini Forzza

**Affiliations:** 1 Instituto de Pesquisas Jardim Botânico do Rio de Janeiro, Rio de Janeiro, Brazil Instituto de Pesquisas Jardim Botânico do Rio de Janeiro Rio de Janeiro Brazil; 2 Instituto de Biologia, Universidade Federal do Rio de Janeiro, Rio de Janeiro, Brazil Instituto de Biologia, Universidade Federal do Rio de Janeiro Rio de Janeiro Brazil

**Keywords:** Caatinga domain, Federal Conservation Units, Flora samples

## Abstract

**Background:**

This article provides a quantitative description of flora specimens stored in the Jardim Botânico of Rio de Janeiro Herbarium that belongs to the Federal Conservation Units of Caatinga’s phytogeography domain. The Caatinga represents 11% of Brazilian territory and is, in South America, the largest and most biodiverse semi-arid tropical ecoregion, yet only 5% of its territory is covered by Federal Conservation Units, with few collections of flora samples. Thus, providing a georeferenced inventory of existing collections is essential for purposes of species distribution, environmental management and conservation. The aim of this data paper is to gauge, by means of geographic coordinates correction and retrieval of the flora specimens present in the RB Herbarium, the amount of specimen gatherings performed in the Federal Conservation Units belonging to the Caatinga domain.

**New information:**

Currently, the RB data is publicly available online at several biodiversity portals, such as our institutional database JABOT, the Reflora Virtual Herbarium, the SiBBr and the GBIF portal (Lanna et al. 2019). However, a description of the dataset that belongs to the Federal Conservation Units of Caatinga’s phytogeography domain as a whole is not yet available in the literature.

## Introduction

The Caatinga phytogeographic domain is South America’s largest and most biodiverse tropical semi-arid ecoregion (Moro et al. 2016). It occupies interplanaltic depression areas northeast and northwards of Minas Gerais state in Brazil (Ab’Saber 2007) and is distributed along an estimated area of 844,000 km². Originally, the biome represented 11% of the Brazilian territory (Ministério do Meio Ambiente 2018). Although the diversity of plants and animals may be considered low when compared with other regions (e.g. tropical forests), the biological patrimony in this region is adapted to the local extreme conditions, resulting in high endemism rates (Queiroz et al. 2017).

Caatinga encompasses woody vegetation that is thorny and deciduous (Ab’Saber 1974) and can be divided into 12 types according to abiotic variations of altitude, continentality and soil features (Alves et al. 2008, Araújo et al. 2005). This biome is facing continuous deforestation processes related to wood extraction, farming and agriculture (Ribeiro et al. 2015, Marinho et al. 2016), which generates negative effects on biodiversity, such as habitat loss and fragmentation (Antongiovanni et al. 2018) and desertification (Hauff 2010).

Conservation Units are defined as territorial spaces protected by public or private initiatives in order to promote biodiversity conservation, restoration and management, as well as to protect natural resources while encompassing extractivism and sustainable uses. In Brazil, the National System of Conservation Units (Brasil 2011) separates Conservation Units into two conservation groups and some categories of preservation. The Full Protection Units group targets nature preservation in a restrictive way, accepting only indirect use of its natural resources. On the other hand, the Sustainable Use Units aim to reconcile nature conservation and sustainable uses.

Less than 5% of Caatinga’s territory is currently covered by Conservation Units (CU) (Oliveira et al. 2013), whereas 30% of its typical biodiversity occurs entirely outside of protected areas, which promotes gaps in the protection, research and management of flora species (Fonseca and Venticinque 2018). It is estimated that at least 4,843 species of plants have been registered for this region (BFG 2018), with 1,523 cases of endemism (Flora do Brasil 2020 2019)

Although the importance and conservation of Caatinga’s biodiversity is well recognised, new gatherings of flora specimens are scarce (Correia et al. 2019, Moro et al. 2016). In this regard, for collected samples, a specimens’ inventory is essential for subsidising systematic analyses that are capable of promoting scientific research projects, public policies and guiding authorities’ decisions about its management and restoration (Silva et al. 2017).

**Figure 1.**
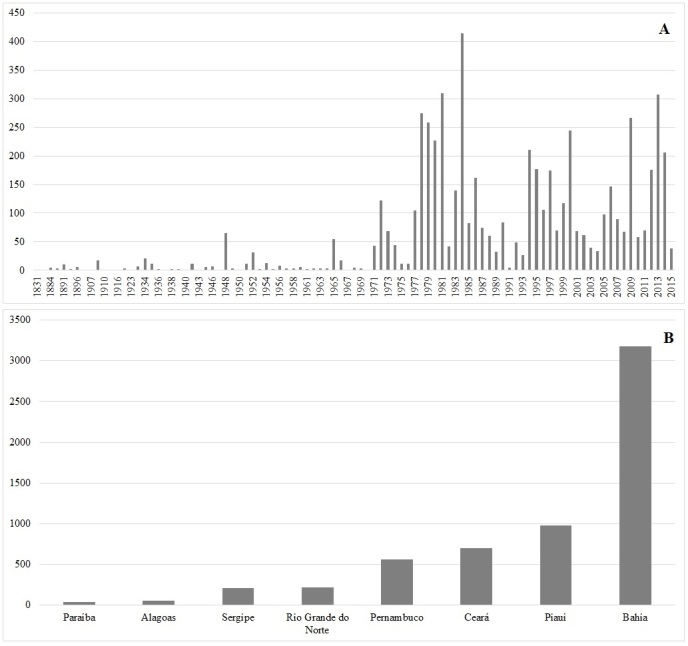
Number of Caatinga’s species deposited in the RB Herbarium. **A.** Number of species deposited per year; **B.** Number of species deposited per State.

The RB Herbarium of Jardim Botânico of Rio de Janeiro (JBRJ), created in 1890, is composed of seven botanical collections: mounted specimens (RB – 750,000, with 7,500 nomenclatural types and ~3,000 paratypes), wood (RBw - ca. 10,300 specimens), fruits (RBcarpo - ca. 8,000 specimens), DNA bank (RBdna - ca. 5,700 specimens), spirit (RBspirit - ca. 2,500 specimens), seedbank (RBsem - ca. 2,700 specimens) and ethnobotany (RBetno – ca. 200 specimens) (Forzza et al. 2016, Lanna et al. 2018). Data used in the present work were acquired in the RB Herbarium collections and exhibit a historical series encompassing approximately 130 years (Fig. 1A), distributed throughout eight states (Fig. 1B).

Our goal is to gauge the amount of collections performed in Federal Conservation Units that belong to the Caatinga domain through the correction and recovery of geographic coordinates of flora samples present in the RB Herbarium. The objective is to make the material available in the Jardim Botânico Botanical Collections Management System (Jabot) (Silva et al. 2017) to assist historical series research development (Kamino et al. 2011), predictive modelling for species distributions and to support conservation status assessments.

## Sampling methods

### Study extent

**Figure 2.**
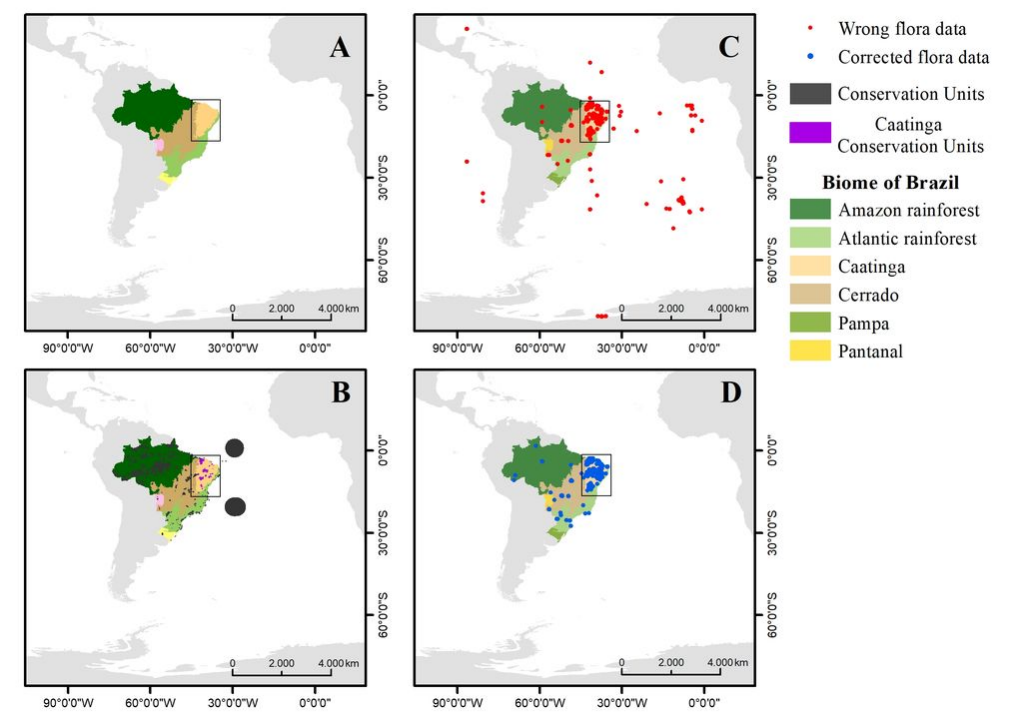
**A.** Study area; **B. **Spatial distribution of Brazilian Conservation Units according to the Brazilian Ministry of the Environment; **C.** Samples’ distribution before geographic coordinates correction; **D.** Samples’ distribution after geographic correction. Maps were prepared using ArcGIS.

The Caatinga biome has 25 Federal Conservation Units that total 31,952 km², which represents 5% of its territory and encompasses the states of Alagoas, Bahia, Ceará, Maranhão, Pernambuco, Paraíba, Rio Grande do Norte, Piauí, Sergipe and the north of Minas Gerais (Brasil 2018). Despite the long collection of historical data (Fig. 1), scarce samples are still in the states of Maranhão and north of Minas Gerais (Fig. 2). One of the main reasons for such scarcity is that these states are territories of transition between the Cerrado and the Caatinga biomes, in which Conservation Units are commonly categorised as belonging to the Cerrado biome. The study area, the Conservation Units spatial distribution and samples of pre- and post-geographic coordinates correction are presented in Fig. 2.

### Sampling description

The determination of the set of samples of Caatinga flora, present in the RB herbarium that would be analysed, was determined through a selection in Jabot, where all the samples existing at the Caatinga domain were acquired. Subsequently, a sub-selection was performed in order to acquire all the samples collected only at the municipalities that present Conservation Units, as illustrated in Fig. 2C. The sample universe encompassed 6,058 data related to 25 Federal Conservation Units (Fig. 2B).

### Quality control

Thereafter, the methodological procedures present in Magdalena et al. (2018) were adopted: (1) definition of geographic reference base; (2) identification of the records with geographic coordinates; (3) identification of the records without geographic coordinates; and (4) location search

The Continuous Cartographic Base of Brazil 1:250,000 (CB250) (Brasil, Coordenação de Cartografia (CCAR), Diretoria de Geociências (DGC) 2017) was the geographic reference base adopted to perform the analyses. This base is provided by the Instituto Brasileiro de Geografia e Estatística (IBGE) and includes all the 5,570 municipalities of the 26 states and one Federal District that constitute the formal divisions of Brazil. Using CB250 allowed the standardisation and synchronisation of geographic names and limits, as well as the coordinates included in the collected samples.

All coordinates were converted to decimal format to validate a record. Then, if they matched, data were marked as correct. If any inconsistencies amongst the data were found, data were investigated to identify the issues regarding them. Two usual types of problems related to geographic data from botanical collections were then analysed: (1) error caused by the reversal of latitude and longitude values and (2) the absence of a cardinal direction that identifies the N–S and E–W hemispheres. The solution for the first case was to invert the coordinates based on the location details. In the second case, it was necessary to denominate the cardinal direction, according to the locality described the record.

Typically, records with geographic coordinates are scarce in historical herbaria, such as RB. Hence, the process previously described should be applied to individually analyse incompatible data. This was necessary due to some errors, such as typing coordinates in an incompatible way, confusing coordinates with regards to international (longitude and latitude) and national (latitude and longitude) standards; typing municipalities that are homonyms but belong to different states (e.g. Bonito, Mato Grosso do Sul and Bonito, Pernambuco).

After establishing records that lack geographic coordinates, a filter was applied to distinguish collections with and without a location description. For the specimen labels that include a location description, analyses were carried out to ensure the greatest accuracy possible when inferring the geographic coordinates. For these data, the user is informed that the stated coordinates were estimated by the description on the specimen label.

It was possible to infer the location of the collection for a record without coordinates if the specimen had a location description and/or some of the following information on the label: country, state, municipality, gazetteer, name of protected area (if informed) and physical toponyms. A georeferencing process was then conducted using toponym lists from IBGE and other federal institutes with database services, such as Serviço Geológico do Brasil, Instituto Chico Mendes de Conservação da Biodiversidade, Instituto Brasileiro do Meio Ambiente e dos Recursos Naturais Renováveis and Ministério do Meio Ambiente - MMA. This is the slowest phase of the process because it is usually carried out record by record. In order to optimise the location, searches of all the collections from the same location were grouped and, thereupon, the coordinates were inferred for the group.

## Geographic coverage

### Description

Results revealed 5,948 samples checked and recovered (98%), of which 2,408 (34%) were located inside Conservation Units’ (CU) limits, 1,383 encompassed the Full Protection Unit category and 1,025 were related to the Sustainable Use Units category (Fig. 2D).

A total of 2,818 (47%) samples were placed in the Buffer Zone (BZ) of 10 km and 722 (12%) were outside of this zone. Regarding the remaining data, 110 (2%) did not present enough information to enable the geographic coordinate’s recovery due to the absence of locality description. Table 1 and Table 2 show the number of samples collected per Conservation Unit category.

### Coordinates

-16.046 and -2.636 Latitude; -34.673 and -44.473 Longitude.

## Collection data

### Collection name

Herbário Dimitri Sucre Benjamin

### Collection identifier

RB

## Usage rights

### Use license

Creative Commons Public Domain Waiver (CC-Zero)

## Data resources

### Data package title

 Caatinga Biome - RB - Rio de Janeiro Botanical Garden Herbarium Collection

### Resource link

https://www.gbif.org/dataset/98d7a49c-5776-41eb-a928-b6f5174eadd1

### Number of data sets

1

### Data set 1.

#### Data set name

Caatinga Biome - RB - Rio de Janeiro Botanical Garden Herbarium Collection

#### Data format

Darwin Core Archive (DwC-A)

#### Number of columns

48

#### Download URL

http://ipt.jbrj.gov.br/jbrj/resource?r=rb_caatinga&v=1.25

#### 

**Data set 1.** 

Column label	Column description
occurrenceID	The unique identifier of the occurrence.
type	The nature or genre of the resource.
modified	The most recent date-time on which the resource was changed.
rightsHolder	The organisation owning or managing rights over the resource.
institutionCode	The name in use by the institution having custody of the object or information referred to in the record.
collectionCode	The name coden or initialism identifying the collection or dataset from which the record was derived.
basisOfRecord	The specific nature of the data record.
catalogNumber	Barcode of the specimen.
occurrenceRemarks	Comments or notes about the occurrence.
recordNumber	The collector's number.
recordedBy	A list of names of people responsible for recording the original occurrence.
otherCatalogNumbers	Sequential register number historically adopted by the RB herbarium.
associatedMedia	A list, concatenated and separated by "|" of the specimens images URLs in a low resolution format to be used as thumbnails.
associatedOccurrences	A list of identifiers of other occurrence records and their associations with this occurrence.
eventDate	Date of collection.
year	Year of collection.
month	Month of collection.
day	Day of collection.
fieldNotes	The text of notes taken in the field about the specimen.
country	The name of the country or major administrative unit in which the Location occurs.
countryCode	The standard code for the country .
stateProvince	The name of the next smaller administrative region than country in which the Location occurs.
locality	The specific description of the place.
municipality	A spatial region or named place.
minimumElevationInMetres	The lower limit of the range of elevation (altitude, usually above sea level), in metres.
maximumElevationInMetres	The upper limit of the range of elevation (altitude, usually above sea level), in metres.
verbatimLatitude	The verbatim original latitude of the Location.
verbatimLongitude	The verbatim original longitude of the Location.
decimalLatitude	The geographic latitude of the geographic centre of a Location.
decimalLongitude	The geographic longitude of the geographic centre of a Location.
geodeticDatum	The ellipsoid, geodetic datum or spatial reference system (SRS) upon which the geographic coordinates given in decimalLatitude and decimalLongitude as based.
georeferencedDate	The date on which the Location was georeferenced.
georeferenceProtocol	A description or reference to the methods used to determine the spatial footprint, coordinates and uncertainties.
georeferenceVerificationStatus	A categorical description of the extent to which the georeference has been verified to represent the best possible spatial description.
georeferenceRemarks	Notes or comments about the spatial description determination.
identifiedBy	A list of names of people, groups or organisations who assigned the Taxon to the subject.
dateIdentified	The date on which the subject was identified as representing the Taxon.
identificationRemarks	Comments or notes about the identification.
identificationQualifier	A standard term to express the determiner's doubts about the identification.
typeStatus	Status of the type. Controlled vocabulary of terms. The category "TYPUS" is used for undefined type status.
scientificName	The full scientific name, with authorship.
kingdom	The full scientific name of the kingdom in which the taxon is classified.
family	The full scientific name of the family in which the taxon is classified.
genus	The full scientific name of the genus in which the taxon is classified.
specificEpithet	The name of the first or species epithet of the scientificName.
infraspecificEpithet	The name of the lowest or terminal infraspecific epithet of the scientificName.
taxonRank	The taxonomic rank of the most specific name in the scientificName.
scientificNameAuthorship	The authorship information for the scientificName.

## Figures and Tables

**Table 1.** Full Protection Units.

Name	Collects on the CU	Collects on the BZ	Total (CU + BZ)
Esec Aiuaba	10	14	24
Esec Castanhão	0	0	0
Esec Seridó	21	7	28
Esec Raso da Catarina	44	34	78
MN Rio São Francisco	94	140	234
Parna Chapada Diamantina	784	1788	2572
Parna Furna Feia	0	27	27
Parna Serra da Capivara	79	237	316
Parna Jericoacoara	0	2	2
Parna Sete Cidades	205	0	205
Parna Ubajara	35	63	98
Parna Catimbau	80	126	206
Rebio Serra Negra	31	30	61
Total	1383	2468	3851

**Table 2.** Sustainable Use Units.

Name	Collects on the CU	Collects on the BZ	Total (CU + BZ)
APA Chapada do Araripe	407	123	530
APA Serra da Ibiapaba	404	66	470
APA Serra da Meruoca	14	13	27
Arie Cocorobó	0	0	0
Arie Vale dos Dinossauros	0	23	23
Flona Contendas do Sincorá	5	32	37
Flona Açu	0	46	46
Flona Negreiros	0	0	0
Flona Palmares	0	9	9
Flona Sobral	16	6	22
Flona Araripe-Apodi	178	28	206
Resex Batoque	1	4	5
Total	1025	350	1375
